# Supplementary material for: Genome-Wide Identification of 2-Oxoglutarate and Fe (II)-Dependent Dioxygenase (2ODD-C) Family Genes and Expression Profiles under Different Abiotic Stresses in Camellia sinensis (L.)
Source: Plants (Basel). 2023 Mar 14;12(6):1302. doi: 10.3390/plants12061302 (PMC10051519; doi:10.3390/plants12061302)
Supplement: Supplementary file 1 [file plants-12-01302-s001.zip › TableS1.pdf]

Table S1 The detail information of CsODD genes in *C. sinensis*

ID	Gene	Chr	Intron	Predicted Locaion	Chromosome position		Protein properties		
					Start	End	Length（aa）	Pl	Mw（Kda）
Csodd-C1	CSS0018726.1	Contig1264	2	Cytoplasm	110608	116401	343	8.01	38.22
Csodd-C2	CSS0030708.1	Chr 11	2	Cytoplasm	3975710	3981383	335	7.69	37.49
Csodd-C3	CSS0021896.1	Chr 5	2	Cytoplasm	192622146	192629165	344	5.81	38.72
Csodd-C4	CSS0029172.1	Chr 8	2	Cytoplasm	3305454	103307027	334	5.43	37.56
Csodd-C5	CSS0001566.1	Chr 9	2	Cytoplasm	3029515	3035878	362	6.27	40.44
Csodd-C6	CSS0000335.1	Chr 7	2	Cytoplasm	34743523	34746076	333	6.16	37.19
Csodd-C7	CSS0002801.1	Chr 7	2	Cytoplasm	34543734	34546926	333	6.16	37.19
Csodd-C8	CSS0010875.1	Chr 5	2	Cytoplasm	33956802	33959389	332	7.88	37.32
Csodd-C9	CSS0034370.1	Chr 1	4	Cytoplasm	148502510	148502737	338	5.97	38.73
Csodd-C10	CSS0032224.1	Chr 1	3	Cytoplasm	111864885	111885954	338	5.97	38.73
Csodd-C11	CSS0044637.1	Chr 7	3	Cytoplasm	183482816	183514115	337	5.27	38.72
Csodd-C12	CSS0003495.1	Chr 12	3	Cytoplasm	134186879	134191842	356	5.14	40.32
Csodd-C13	CSS0042033.1	Chr 7	3	Cytoplasm	183414418	183437718	337	5.44	38.72
Csodd-C14	CSS0007535.1	Chr 9	3	Cytoplasm	10504217	10507942	365	8.2	40.06
Csodd-C15	CSS0014892.1	Chr 9	3	Cytoplasm	9294053	9297535	365	8.2	40.11
Csodd-C16	CSS0033075.1	Chr 4	2	Cytoplasm	67261223	67266397	331	5.79	37.56
Csodd-C17	CSS0046529.1	Contig1134	2	Cytoplasm	738397	744362	331	5.43	37.59
Csodd-C18	CSS0021865.2	Chr 4	2	Cytoplasm	166079410	166089721	341	6.74	39.46
Csodd-C19	CSS0011972.1	Chr 2	1	Cytoplasm	116058917	116060956	373	8.25	41.08
Csodd-C20	CSS0047148.1	Chr 2	1	Cytoplasm	115965632	115967404	373	8.25	41.08
Csodd-C21	CSS0046316.1	Chr 3	2	Cytoplasm	13398596	13402483	351	5.46	40.08
Csodd-C22	CSS0030401.1	Chr 9	2	Cytoplasm	59599291	59603560	334	6.44	37.89
Csodd-C23	CSS0036541.1	Chr 15	1	Cytoplasm	25894721	25897043	361	7.18	40.32
Csodd-C24	CSS0009911.1	Chr 14	3	Cytoplasm	34381490	34384961	365	4.88	40.86
Csodd-C25	CSS0006465.1	Chr 15	1	Cytoplasm	25409475	25411622	361	7.18	40.23
Csodd-C26	CSS0011888.1	Chr 10	2	Cytoplasm	137637759	137643274	362	6.51	40.81
Csodd-C27	CSS0003382.1	Chr 13	3	Cytoplasm	80058876	80064037	341	6.44	38.93
Csodd-C28	CSS0030853.1	Chr 3	3	Cytoplasm	13355548	13358628	333	6.3	38.7
Csodd-C29	CSS0008117.1	Chr 5	2	Cytoplasm	133842097	133845493	377	6.8	42.59
Csodd-C30	CSS0019461.1	Chr 1	2	Cytoplasm	75597351	75599523	383	6.64	43
Csodd-C31	CSS0009584.1	Chr 5	2	Cytoplasm	137818203	137821604	377	6.72	42.71
Csodd-C32	CSS0007745.1	Chr 9	2	Cytoplasm	59548673	59560708	336	5.17	38.4
Csodd-C33	CSS0019623.1	Chr 1	2	Cytoplasm	159550132	159553332	378	7.36	43.04
Csodd-C34	CSS0008358.1	Chr 8	2	Cytoplasm	120988611	120996669	332	6.51	38.31
Csodd-C35	CSS0010021.1	Chr 8	3	Cytoplasm	70912531	70935552	361	5.06	41.22
Csodd-C36	CSS0006235.1	Chr 13	3	Cytoplasm	80362542	80367823	341	6.04	38.83
Csodd-C37	CSS0008107.1	Chr 9	2	Cytoplasm	145014231	145017595	373	6.34	42.23
Csodd-C38	CSS0009475.1	Chr 9	2	Cytoplasm	129899680	129903064	375	7.48	42.61

Csodd-C39	CSS0035145.1	Chr 1	3	Cytoplasm	18028988	18032759	367	6.35	41.65
Csodd-C40	CSS0030656.1	Chr 9	2	Cytoplasm	137530916	137534428	374	6.23	42.31
Csodd-C41	CSS0001763.1	Chr 1	3	Cytoplasm	125813218	125816934	342	5.83	38.61
Csodd-C42	CSS0037910.1	Chr 4	1	Cytoplasm	157096232	157096786	341	6.95	38.9
Csodd-C43	CSS0044103.1	Chr 7	2	Cytoplasm	15997144	16001372	333	6.17	37.91
Csodd-C44	CSS0007481.1	Chr 4	2	Cytoplasm	185704870	185710799	359	6.51	40.45
Csodd-C45	CSS0017409.1	Chr 4	2	Cytoplasm	57036013	57040804	376	7.42	43.02
Csodd-C46	CSS0045924.1	Chr 4	2	Cytoplasm	67017586	67026144	339	5.11	38.82
Csodd-C47	CSS0011799.1	Chr 14	2	Cytoplasm	93409064	93421650	323	9.55	36.56
Csodd-C48	CSS0009221.1	Chr 7	2	Cytoplasm	143917051	143929702	374	6.94	41.84
Csodd-C49	CSS0031283.1	Contig1189	2	Cytoplasm	1680	10870	363	6.37	40.82
Csodd-C50	CSS0019497.1	Chr 1	2	Cytoplasm	40911835	40917704	363	6.51	40.8
Csodd-C51	CSS0042210.1	Chr 10	3	Cytoplasm	99959571	99963481	390	6.38	44.45
Csodd-C52	CSS0044406.1	Chr 11	3	Cytoplasm	88941199	88942964	340	4.71	38.84
Csodd-C53	CSS0039029.1	Chr 14	2	Cytoplasm	94751537	94766725	323	9.25	36.5
Csodd-C54	CSS0036121.1	Chr 4	2	Cytoplasm	159515000	159519413	366	6.07	41.34
Csodd-C55	CSS0019378.1	Chr 9	2	Cytoplasm	22664785	22666758	378	6.05	42.42
Csodd-C56	CSS0028732.1	Chr 13	3	Cytoplasm	79326831	79328302	341	6.67	38.08
Csodd-C57	CSS0016177.1	Chr 9	2	Cytoplasm	149171408	149175923	368	5.56	41.47
Csodd-C58	CSS0032063.1	Chr 5	2	Cytoplasm	18816392	18819594	328	5.99	37.44
Csodd-C59	CSS0039460.1	Chr 8	2	Cytoplasm	17404712	17411024	335	5.16	38.54
Csodd-C60	CSS0008204.1	Chr 8	2	Cytoplasm	158484243	158487327	358	5.92	39.83
Csodd-C61	CSS0042979.1	Chr 11	3	Cytoplasm	88952540	88954934	333	5.23	36.97
Csodd-C62	CSS0006299.1	Chr 12	1	Cytoplasm	30285858	30296451	358	5.62	40.14
Csodd-C63	CSS0019002.1	Chr 1	2	Cytoplasm	172500388	172503877	356	5.01	39.93
Csodd-C64	CSS0022476.1	Chr 7	3	Cytoplasm	43386826	43389680	320	5.29	36.21
Csodd-C65	CSS0007851.1	Chr 15	2	Cytoplasm	88513452	88519310	323	5.1	36.38
Csodd-C66	CSS0018017.1	Chr 5	2	Cytoplasm	162773981	162778835	378	6.32	42.38
Csodd-C67	CSS0031289.1	Chr 8	3	Cytoplasm	7380071	7383157	318	4.97	36.27
Csodd-C68	CSS0028530.1	Chr 13	3	Cytoplasm	79389481	79391158	345	5.78	38.59
Csodd-C69	CSS0000425.1	Chr 4	2	Cytoplasm	60509082	60511600	312	5.09	35.53
Csodd-C70	CSS0027544.1	Contig127	2	Cytoplasm	4061	6903	312	5.09	35.53
Csodd-C71	CSS0024933.1	Chr 5	2	Cytoplasm	163228421	163231372	371	5.4	41.75
Csodd-C72	CSS0031656.1	Chr 6	2	Cytoplasm	45315518	45318390	306	6.25	38.34
Csodd-C73	CSS0047624.1	Chr 3	3	Cytoplasm	33235296	33238103	378	6.98	42.39
Csodd-C74	CSS0034471.1	Contig1013	2	Cytoplasm	163868	166630	312	5.09	35.52
Csodd-C75	CSS0004426.1	Chr9	3	Cytoplasm	89490805	89516404	270	6.96	30.49
Csodd-C76	CSS0020403.1	Chr 5	2	Cytoplasm	37056687	37077502	300	6.28	33.84
Csodd-C77	CSS0003417.1	Chr 13	3	Cytoplasm	79458653	79460167	336	5.21	37.66
Csodd-C78	CSS0000229.1	Chr 1	2	Cytoplasm	153578284	153588879	310	5.22	35.26
Csodd-C79	CSS0024723.1	Chr 13	4	Cytoplasm	79359028	79360383	330	5.57	37.02
Csodd-C80	CSS0041714.1	Chr 8	1	Cytoplasm	71243130	71250682	165	5.01	18.65
Csodd-C81	CSS0048861.1	Chr 7	2	Cytoplasm	62415661	62420865	314	4.56	35.76

Csodd-C82	CSS0041600.1	Chr 7	2	Cytoplasm	62560119	62563575	313	4.8	35.73
Csodd-C83	CSS0041090.1	Chr 1	2	Cytoplasm	153203249	153213796	294	5.57	33.67
Csodd-C84	CSS0050328.1	Chr 5	2	Cytoplasm	163425010	163427853	371	5.82	41.69
Csodd-C85	CSS0016518.1	Chr 5	2	Cytoplasm	163315283	163321801	371	6.19	41.71
Csodd-C86	CSS0015129.1	Chr 11	1	Cytoplasm	107597311	107606838	373	5.93	41.79
Csodd-C87	CSS0031730.1	Chr 4	2	Cytoplasm	133498136	133502273	353	7.29	40.11
Csodd-C88	CSS0036790.1	Chr 4	3	Cytoplasm	133445168	133452526	351	7.07	39.69
Csodd-C89	CSS0010687.1	Chr 14	1	Cytoplasm	50370852	50372936	365	5.65	40.06
Csodd-C90	CSS0016175.1	Chr 8	2	Cytoplasm	163002949	163003882	310	5.67	35.34
Csodd-C91	CSS0006729.1	Chr 7	4	Cytoplasm	3656188	3665223	343	5.81	38.01
Csodd-C92	CSS0013094.1	Chr 9	2	Cytoplasm	22720052	22722840	359	6.3	40.83
Csodd-C93	CSS0031308.1	Chr 2	2	Cytoplasm	78165049	78186020	315	5.41	35.67
Csodd-C94	CSS0029211.1	Chr 12	1	Cytoplasm	144150965	144154281	365	4.96	40
Csodd-C95	CSS0023813.1	Contig1209	2	Cytoplasm	114716	117399	284	6.42	32.32
Csodd-C96	CSS0030231.1	Chr 6	1	Cytoplasm	90284204	90286006	324	4.8	36.5
Csodd-C97	CSS0028241.1	Chr 5	2	Cytoplasm	162729899	162733688	361	5.42	40.1
Csodd-C98	CSS0014098.1	Chr 14	10	Cytoplasm	114704029	114710160	335	4.93	38.54
Csodd-C99	CSS0037766.1	Chr 15	2	Cytoplasm	25930537	25934792	314	5.64	35.66
Csodd-C100	CSS0037970.1	Chr 15	2	Cytoplasm	25435804	25440032	314	5.88	37.75
Csodd-C101	CSS0030295.1	Chr 5	3	Cytoplasm	4262979	4265576	313	5.28	35.68
Csodd-C102	CSS0013741.1	Chr 9	2	Cytoplasm	22356320	22358550	371	5.62	41.89
Csodd-C103	CSS0028792.1	Chr 14	3	Cytoplasm	119061416	119065279	355	4.91	40.72
Csodd-C104	CSS0036983.1	Chr 8	4	Cytoplasm	139595317	139600920	422	6.45	47.36
Csodd-C105	CSS0004829.1	Chr 10	4	Cytoplasm	98799393	98818425	292	4.89	33.18
Csodd-C106	CSS0030637.1	Chr 14	4	Cytoplasm	118976216	118980132	332	6.68	37.45
Csodd-C107	CSS0041639.1	Contig914	2	Cytoplasm	101317	103524	329	6.18	37.24
Csodd-C108	CSS0002912.1	Chr 14	10	Cytoplasm	84592522	84600531	337	5.28	38.74
Csodd-C109	CSS0007997.1	Chr 1	1	Cytoplasm	89451272	89452562	317	6.98	35.82
Csodd-C110	CSS0046216.1	Chr 12	1	Cytoplasm	144124181	144126467	352	5.25	39.95
Csodd-C111	CSS0011056.1	Chr 14	10	Cytoplasm	114442156	114448906	352	5.25	39.95
Csodd-C112	CSS0001975.1	Chr 5	2	Cytoplasm	35613720	35618688	305	5.59	33.94
Csodd-C113	CSS0002044.1	Chr 11	2	Cytoplasm	71377198	71382622	352	7.11	39.38
Csodd-C114	CSS0031464.1	Chr 3	6	Cytoplasm	167370321	167372227	313	5.4	35.83
Csodd-C115	CSS0014812.1	Chr 9	2	Cytoplasm	2489121	22491543	360	6.39	40.67
Csodd-C116	CSS0002163.1	Chr 5	4	Cytoplasm	65930498	65930587	321	4.92	36.67
Csodd-C117	CSS0008883.1	Chr 5	3	Cytoplasm	65815053	65817295	321	4.92	36.67
Csodd-C118	CSS0030701.1	Chr 8	9	Cytoplasm	113610172	113636645	269	4.91	31.14
Csodd-C119	CSS0032638.1	Chr 9	3	Cytoplasm	22449932	22470696	354	6.38	39.79
Csodd-C120	CSS0009643.1	Chr 3	2	Cytoplasm	146882043	146886536	314	6.32	35.98
Csodd-C121	CSS0029177.1	Chr 1	377	Cytoplasm	22449932	22470696	311	6.23	34.56
Csodd-C122	CSS0034241.1	Chr 15	7	Cytoplasm	25533479	25541336	313	6.24	35.89
Csodd-C123	CSS0047673.1	Chr 10	9	Cytoplasm	60446947	60472665	337	5.04	38.67
Csodd-C124	CSS0039776.1	Chr 15	2	Cytoplasm	26024338	26026745	313	6.38	35.88

Csodd-C125	CSS0039676.1	Chr 1	2	Cytoplasm	166134112	166151509	316	6.84	35.45
Csodd-C126	CSS0045512.1	Chr 13	10	Cytoplasm	113368518	113375229	325	5.02	37.21
Csodd-C127	CSS0012096.1	Chr 3	2	Cytoplasm	147064910	147067631	313	5.6	35.82
Csodd-C128	CSS0016828.1	Chr 3	2	Cytoplasm	26024338	26026745	313	5.6	35.82
Csodd-C129	CSS0033274.1	Chr 13	2	Cytoplasm	79334288	79335199	162	8.5	17.53
Csodd-C130	CSS0037947.1	Chr 15	2	Cytoplasm	25954387	25957010	314	4.18	35.61
Csodd-C131	CSS0005236.1	Chr 15	4	Cytoplasm	25464771	25467644	314	6.18	35.61
Csodd-C132	CSS0008939.1	Chr 2	3	Cytoplasm	107302939	107307995	368	4.93	41.59
Csodd-C133	CSS0026038.1	Chr 9	3	Cytoplasm	100715634	100728952	359	6.29	40.5
Csodd-C134	CSS0014460.1	Chr 14	11	Cytoplasm	42377575	42389572	325	4.97	36.54
Csodd-C135	CSS0048328.1	Chr 14	11	Cytoplasm	114805455	114812183	331	5.14	37.9
Csodd-C136	CSS0038898.1	Chr 14	10	Cytoplasm	84542666	84549087	345	6.35	39.85
Csodd-C137	CSS0039070.1	Chr 15	2	Cytoplasm	25941753	25945083	311	5.21	35.47
Csodd-C138	CSS0049117.2	Chr 10	10	Cytoplasm	60533863	60551483	360	6.85	40.73
Csodd-C139	CSS0001124.1	Chr 15	2	Cytoplasm	25449844	25453050	311	5.56	35.49
Csodd-C140	CSS0029347.1	Chr 1	3	Cytoplasm	166223004	166226595	293	6.17	32.3
Csodd-C141	CSS0045784.1	Chr 8	4	Cytoplasm	139525743	139530954	371	5.85	41.27
Csodd-C142	CSS0004808.1	Chr 14	4	Cytoplasm	115290494	115297154	331	4.93	37.71
Csodd-C143	CSS0027842.1	Chr 2	9	Cytoplasm	33092154	33097821	273	5	30.28
Csodd-C144	CSS0044777.1	Chr 15	2	Cytoplasm	25948042	25949857	314	5.12	36.12
Csodd-C145	CSS0001495.1	Chr 14	11	Cytoplasm	42528175	42533095	282	5.17	31.52
Csodd-C146	CSS0013328.1	Chr 14	10	Cytoplasm	42423451	42430641	313	6.24	35.72
Csodd-C147	CSS0049343.1	Chr 9	3	Cytoplasm	101337744	101342352	343	6.58	38.61
Csodd-C148	CSS0020124.1	Chr 15	3	Cytoplasm	25456082	25457775	297	4.9	34.2
Csodd-C149	CSS0009248.1	Chr 5	2	Cytoplasm	163388666	163408780	190	5.06	20.67
Csodd-C150	CSS0031074.1	Chr 9	4	Cytoplasm	63521954	63523856	278	4.82	31.82
Csodd-C151	CSS0018498.1	Chr 3	4	Cytoplasm	25946119	25947865	298	7.29	33.29
Csodd-C152	CSS0028720.1	Chr 2	2	Cytoplasm	107699160	107712158	308	4.34	34.24
Csodd-C153	CSS0024206.1	Chr 6	3	Cytoplasm	112024767	112031124	278	4.64	31.45
