# Supplementary material for: Genome-Wide Identification of 2-Oxoglutarate and Fe (II)-Dependent Dioxygenase (2ODD-C) Family Genes and Expression Profiles under Different Abiotic Stresses in Camellia sinensis (L.)
Source: Plants (Basel). 2023 Mar 14;12(6):1302. doi: 10.3390/plants12061302 (PMC10051519; doi:10.3390/plants12061302)
Supplement: Supplementary file 1 [file plants-12-01302-s001.zip › TableS10.pdf]

Table S10 The primer sequences used in this study

Primer ID	Sequence	Primer ID	Sequence
CsTBP-F	GGCGGATCAAGTGTTGGAAGGGAG	CsODD-C72-F	GCAGACAAGGGCCCCAGATTTG
CsTBP-R	ACGCTTGGGATTGTATTCGGCATTA	CsODD-C72-R	GAAGCCCCAGTCTGCACAAGCC
CsODD-C8-F	GTCGAATATCTCCTCCTAAC	CsODD-C80-F	TCAATGGAGTTGAGGGCTTC
CsODD-C8-R	TAACTCTAGAACCTCACAAG	CsODD-C80-R	TGCTCTAAGAATGACTTTCCGT
CsODD-C27-F	GTAGCATCAGAGTTAATGAAGG	CsODD-C95-F	GCTGGTGGAATCATTCTCTTGC
CsODD-C27-R	CCTTCTCATTGGTATAGGTTAG	CsODD-C95-R	ATAGCATTGAGCACAGAGCA
CsODD-C35-F	TGTCCAATTCTCTCAGAAGATG	CsODD-C107-F	TGCTCGGATAGCTCCTTTCTA
CsODD-C35-R	CAGTTTCTCCACTAGTAAAGGG	CsODD-C107-R	AGAGGTTGAGAGACTTGAGGAT
CsODD-C36-F	CCAGAATCAGTTGATGAGTA	CsODD-C109-F	CTCTCTACAACTTCCTGTAT
CsODD-C36-R	CTTGGTTAAGAAGGGTAAGA	CsODD-C109-R	CCATACTCTCTCAGTATCTC
CsODD-C39-F	GGAGAGACTTCTTGAAGCTTAG	CsODD-C121-F	GCTTTTCGTTTGAGGGATGGCC
CsODD-C39-R	CCTTAAGTCAGAAGGAGAAGAA	CsODD-C121-R	GAAAGGAACATACAAGCGTGAG
CsODD-C44-F	CTTCTTCTACAATCCCAATG		
CsODD-C44-R	CCTTGTCCTAATGAACAATC		
