# Supplementary material for: Genome-Wide Identification of 2-Oxoglutarate and Fe (II)-Dependent Dioxygenase (2ODD-C) Family Genes and Expression Profiles under Different Abiotic Stresses in Camellia sinensis (L.)
Source: Plants (Basel). 2023 Mar 14;12(6):1302. doi: 10.3390/plants12061302 (PMC10051519; doi:10.3390/plants12061302)
Supplement: Supplementary file 1 [file plants-12-01302-s001.zip › TableS2.pdf]

Table S2 Segmentally duplicated *Cs2ODD-C* gene pairs.

Seq_1	Gene_ID_1	Seq_2	Gene_ID_2	Ka	Ks	Ka/Ks
Csodd-C2	CSS0030708	Csodd-C3	CSS0021896	0.2761	1.6756	0.1648
Csodd-C3	CSS0021896	Csodd-C5	CSS0001566	0.1313	0.5269	0.2493
Csodd-C10	CSS0032224	Csodd-C13	CSS0042033	0.065	0.7707	0.0843
Csodd-C23	CSS0036541	Csodd-C20	CSS0047148	0.1059	0.5861	0.1806
Csodd-C25	CSS0006465	Csodd-C20	CSS0047148	0.1059	0.5694	0.1859
Csodd-C26	CSS0011888	Csodd-C44	CSS0007481	0.1422	0.6297	0.2259
Csodd-C28	CSS0030853	Csodd-C42	CSS0037910	0.2150	1.2972	0.1658
Csodd-C44	CSS0007481	Csodd-C48	CSS0009221	0.2157	1.8854	0.1144
Csodd-C46	CSS0045924	Csodd-C32	CSS0007745	0.1075	0.7166	0.1500
Csodd-C50	CSS0019497	Csodd-C26	CSS0011888	0.2856	2.5541	0.1118
Csodd-C52	CSS0044406	Csodd-C56	CSS0028732	0.3132	1.2473	0.2511
Csodd-C59	CSS0039460	Csodd-C34	CSS0008358	0.1057	0.4429	0.2387
Csodd-C64	CSS0022476	Csodd-C67	CSS0031289	0.1211	0.9414	0.1286
Csodd-C65	CSS0007851	Csodd-C93	CSS0031308	0.2731	1.5087	0.1810
Csodd-C105	CSS0004829	Csodd-C69	CSS0000425	0.0117	0.0272	0.4298
Csodd-C110	CSS0046216	Csodd-C89	CSS0010687	0.0946	0.8659	0.1092
Csodd-C113	CSS0002044	Csodd-C88	CSS0036790	0.2073	0.7531	0.2753
Csodd-C117	CSS0008883	Csodd-C64	CSS0022476	0.0677	0.4952	0.1366
Csodd-C5	CSS0001566	Csodd-C2	CSS0030708	0.2637	1.3641	0.1933
Csodd-C44	CSS0007481	Csodd-C50	CSS0019497	0.2144	1.8284	0.1173
Csodd-C48	CSS0009221	Csodd-C50	CSS0019497	0.1341	1.1475	0.1168
Csodd-C67	CSS0031289	Csodd-C117	CSS0008883	0.1182	0.9171	0.1289
Csodd-C102	CSS0013741	Csodd-C71	CSS0024933	0.3129	1.4492	0.2159
