# Supplementary material for: Genome-Wide Identification of 2-Oxoglutarate and Fe (II)-Dependent Dioxygenase (2ODD-C) Family Genes and Expression Profiles under Different Abiotic Stresses in Camellia sinensis (L.)
Source: Plants (Basel). 2023 Mar 14;12(6):1302. doi: 10.3390/plants12061302 (PMC10051519; doi:10.3390/plants12061302)
Supplement: Supplementary file 1 [file plants-12-01302-s001.zip › TableS3.pdf]

Table S3 One-to-one orthologous relationships between *C. sinensis* and *Arabidopsis*.

Seq_1	Gene_ID_1	Seq_2	Gene_ID_2	Ka	Ks	Ka/Ks
CsODD-C2	CSS0030708	AtODD-C1	AT1G02400	0.3161	4.1659	0.0759
CsODD-C3	CSS0021896	AtODD-C22	AT1G47990	0.3477	4.3139	0.0806
CsODD-C5	CSS0001566	AtODD-C22	AT1G47990	0.3931	5.1588	0.0762
CsODD-C10	CSS0032224	AtODD-C62	AT4G10490	0.3620	4.7569	0.0761
CsODD-C13	CSS0042033	AtODD-C80	AT5G24530	0.2271	2.8841	0.0787
CsODD-C14	CSS0007535	AtODD-C50	AT3G21420	0.2301	2.0095	0.1145
CsODD-C20	CSS0047148	AtODD-C35	AT1G80330	0.3899	3.0095	0.1040
CsODD-C23	CSS0036541	AtODD-C35	AT1G80330	0.4265	5.1259	0.0832
CsODD-C25	CSS0006465	AtODD-C15	AT1G15550	0.3285	5.6508	0.0581
CsODD-C26	CSS0011888	AtODD-C43	AT2G38240	0.3264	4.3478	0.0751
CsODD-C28	CSS0030853	AtODD-C48	AT3G19000	0.287	3.5291	0.0813
CsODD-C30	CSS0019461	AtODD-C85	AT5G51810	0.2657	3.3943	0.0783
CsODD-C48	CSS0009221	AtODD-C43	AT2G38240	0.3273	3.8175	0.0857
CsODD-C50	CSS0019497	AtODD-C58	AT3G55970	0.2753	2.7909	0.0986
CsODD-C59	CSS0039460	AtODD-C66	AT4G21200	0.2406	3.2357	0.0744
CsODD-C67	CSS0031289	AtODD-C11	AT1G12010	0.1876	1.6284	0.1152
CsODD-C69	CSS0000425	AtODD-C31	AT1G77330	0.1797	1.9480	0.0922
CsODD-C72	CSS0031656	AtODD-C37	AT2G19590	0.1808	2.0422	0.0885
CsODD-C88	CSS0036790	AtODD-C53	AT3G47190	0.4820	2.6213	0.1839
CsODD-C91	CSS0006729	AtODD-C46	AT3G12900	0.2967	2.243	0.1323
CsODD-C99	CSS0037766	AtODD-C25	AT1G52790	0.4454	1.9477	0.2287
CsODD-C106	CSS0030637	AtODD-C78	AT5G20400	0.4198	2.4746	0.1696
CsODD-C109	CSS0007997	AtODD-C69	AT4G23340	0.2478	2.8916	0.0857
CsODD-C113	CSS0002044	AtODD-C87	AT5G58660	0.4376	1.8694	0.2341
CsODD-C117	CSS0008883	AtODD-C6	AT1G05010	0.1630	2.4506	0.0665
CsODD-C118	CSS0030701	AtODD-C30	AT1G62380	0.2289	1.7153	0.1335
CsODD-C137	CSS0039070	AtODD-C34	AT1G80320	0.5797	4.1297	0.1404
CsODD-C139	CSS0001124	AtODD-C34	AT1G80320	0.5795	3.6352	0.1594
CsODD-C148	CSS0020124	AtODD-C14	AT1G15540	0.4914	2.6642	0.1844
CsODD-C149	CSS0009248	AtODD-C82	AT5G43450	0.4761	3.8274	0.1244
CsODD-C150	CSS0031074	AtODD-C31	AT1G77330	0.1923	2.9432	0.0653
CsODD-C117	CSS0008883	AtODD-C11	AT1G12010	0.1887	1.8324	0.1030
CsODD-C67	CSS0031289	AtODD-C30	AT1G62380	0.2068	2.0008	0.1034
CsODD-C50	CSS0019497	AtODD-C45	AT3G11180	0.2776	2.1027	0.1320
CsODD-C50	CSS0019497	AtODD-C73	AT5G05600	0.2288	1.1603	0.1972
CsODD-C10	CSS0032224	AtODD-C80	AT5G24530	0.2247	1.1573	0.1942
