# Supplementary material for: Genome-Wide Identification of 2-Oxoglutarate and Fe (II)-Dependent Dioxygenase (2ODD-C) Family Genes and Expression Profiles under Different Abiotic Stresses in Camellia sinensis (L.)
Source: Plants (Basel). 2023 Mar 14;12(6):1302. doi: 10.3390/plants12061302 (PMC10051519; doi:10.3390/plants12061302)
Supplement: Supplementary file 1 [file plants-12-01302-s001.zip › TableS4.pdf]

Table S4 One-to-one orthologous relationships between *C. sinensis* and rice.

Seq_1	Gene_ID_1	Seq_2	Gene_ID_2	Ka	Ks	Ka/Ks
CsODD-C14	CSS0007535	OsODD-C12	LOC_Os01g70930	0.3361	2.9067	0.1156
CsODD-C23	CSS0036541	OsODD-C1	LOC_Os01g08220	0.5519	2.7090	0.2037
CsODD-C26	CSS0011888	OsODD-C18	LOC_Os03g18030	0.3551	2.1526	0.1650
CsODD-C44	CSS0007481	OsODD-C18	LOC_Os03g18030	0.3414	4.8755	0.0700
CsODD-C48	CSS0009221	OsODD-C18	LOC_Os03g18030	0.3278	2.5327	0.1294
