# Supplementary material for: Genome-Wide Identification of 2-Oxoglutarate and Fe (II)-Dependent Dioxygenase (2ODD-C) Family Genes and Expression Profiles under Different Abiotic Stresses in Camellia sinensis (L.)
Source: Plants (Basel). 2023 Mar 14;12(6):1302. doi: 10.3390/plants12061302 (PMC10051519; doi:10.3390/plants12061302)
Supplement: Supplementary file 1 [file plants-12-01302-s001.zip › TableS5.pdf]

Table S5 The same expression pattern of *CsODD-C* genes under MeJA and PEG treatments

Type	Genes	MeJA			PEG		
		**0 h**	**24 h**	**48 h**	**0 h**	**24 h**	**48 h**
**Type 1**	CsODD-C125	0.26	2.73	4.27	0.38	3.65	2.92
	CsODD-C36	1.09	4.85	3.08	12.98	116.03	58.71
	CsODD-C8	1.20	3.26	2.90	0.66	4.62	5.12
**Type 2**	CsODD-C2	0.22	0.31	0.24	0.41	9.24	1.06
	CsODD-C4	0.00	0.03	0.00	0.00	0.20	0.08
	CsODD-C42	0.00	0.33	0.00	0.00	0.24	0.00
	CsODD-C46	72.74	161.36	63.83	1.27	2.38	0.63
	CsODD-C66	0.10	0.65	0.37	0.53	9.75	2.21
	CsODD-C107	0.00	0.04	0.00	0.00	1.17	0.23
**Type 3**	CsODD-C93	3.29	1.25	19.39	0.00	0.00	0.11

Note: Type1, the expression level of CsODD-C genes were increased after MeJA and PEG treatments; type2, the expression level of CsODD-C genes were increased from 0 h to 24h and then decreased from 24 h to 48 h after MeJA and PEG treatments; type3, CsODD-C genes were only upregulated in 48 h after MeJA and PEG treatments
