# Supplementary material for: Genome-Wide Identification of 2-Oxoglutarate and Fe (II)-Dependent Dioxygenase (2ODD-C) Family Genes and Expression Profiles under Different Abiotic Stresses in Camellia sinensis (L.)
Source: Plants (Basel). 2023 Mar 14;12(6):1302. doi: 10.3390/plants12061302 (PMC10051519; doi:10.3390/plants12061302)
Supplement: Supplementary file 1 [file plants-12-01302-s001.zip › TableS6.pdf]

Table S6 The same expression pattern of *CsODD-C* genes under MeJA and NaCl treatments

Type	Genes	MeJA			NaCL		
		**0 h**	**24 h**	**48 h**	**0 h**	**24 h**	**48 h**
**Type 1**	CsODD-C27	0.05	0.99	0.66	16.54	109.44	79.96
	CsODD-C36	1.09	4.85	3.08	12.98	116.03	58.71
**Type 2**	CsODD-C4	0.00	0.03	0.00	0.00	0.20	0.08
	CsODD-C30	4.97	10.08	3.27	90.27	11.59	40.79
	CsODD-C72	0.21	0.28	0.05	2.61	7.26	4.71
	CsODD-C95	0.21	0.28	0.05	2.61	7.26	4.71
**Type 3**	CsODD-C52	4.40	4.24	5.13	3.13	1.82	2.64
	CsODD-C67	129.25	69.23	249.34	73.46	93.62	70.15
	CsODD-C116	40.78	40.19	99.73	33.56	502.78	153.27
	CsODD-C117	40.78	40.19	99.73	33.56	502.78	153.27

Note: Type1, the expression level of CsODD-C genes were increased after MeJA and PEG treatments; type2, the expression level of CsODD-C genes were increased from 0 h to 24h and then decreased from 24 h to 48 h after MeJA and PEG treatments; type3, CsODD-C genes were only upregulated in 48 h after MeJA and PEG treatments.
