# Supplementary material for: Genome-Wide Identification of 2-Oxoglutarate and Fe (II)-Dependent Dioxygenase (2ODD-C) Family Genes and Expression Profiles under Different Abiotic Stresses in Camellia sinensis (L.)
Source: Plants (Basel). 2023 Mar 14;12(6):1302. doi: 10.3390/plants12061302 (PMC10051519; doi:10.3390/plants12061302)
Supplement: Supplementary file 1 [file plants-12-01302-s001.zip › TableS7.pdf]

Table S7 The same expression pattern of CsODD-C genes under PEG and NaCl treatments.

Type	Genes	PEG			NaCL		
		0 h	24 h	48 h	0 h	24 h	48 h
Type 1	CsODD-C117	33.56	502.78	153.27	33.56	112.36	194.06
	CsODD-C140	1.13	5.86	5.06	1.13	6.13	7.78
	CsODD-C35	0.00	0.37	0.59	0.00	0.55	0.25
	CsODD-C36	12.98	116.03	58.71	12.98	93.77	66.43
	CsODD-C39	0.70	12.05	10.54	0.70	11.85	7.46
	CsODD-C44	2.79	33.43	32.18	2.79	70.32	56.16
	CsODD-C121	16.24	61.81	40.93	16.24	62.41	77.53
	CsODD-C6	2.61	17.10	71.73	2.61	29.22	16.69
Type 2	CsODD-C48	3.41	4.55	0.95	3.41	11.05	2.37
	CsODD-C110	0.00	0.33	0.00	0.00	0.30	0.00
Type 3	-	-	-	-	-	-	-
Type 4	CsODD-C101	11.03	2.04	1.85	11.03	2.66	3.20
	CsODD-C109	0.16	0.00	0.00	0.16	0.00	0.00
	CsODD-C111	13.05	4.58	7.10	13.05	7.90	7.53
	CsODD-C118	13.72	2.40	5.18	13.72	1.28	1.15
	CsODD-C123	12.51	5.67	8.76	12.51	6.03	7.98
	CsODD-C126	13.05	4.58	7.10	13.05	7.90	7.53
	CsODD-C132	106.23	23.13	26.52	106.23	43.79	32.89
	CsODD-C135	13.05	4.58	7.10	13.05	7.90	7.53
	CsODD-C138	12.51	5.67	8.76	12.51	6.03	7.98
	CsODD-C141	266.94	26.22	72.38	266.94	116.76	191.04
	CsODD-C142	13.05	4.58	7.10	13.05	7.90	7.53
	CsODD-C143	7.52	4.29	5.18	7.52	7.15	7.33
	CsODD-C144	0.06	0.00	0.00	0.06	0.00	0.00
	CsODD-C148	0.06	0.00	0.00	0.06	0.00	0.00
	CsODD-C152	106.23	23.13	26.52	106.23	43.79	32.89
	CsODD-C20	0.60	0.21	0.26	0.60	0.13	0.36
	CsODD-C21	418.27	163.19	100.93	418.27	110.97	180.80
	CsODD-C23	0.34	0.00	0.00	0.34	0.00	0.00
	CsODD-C24	1.34	0.28	0.00	1.34	0.33	0.00
	CsODD-C25	0.34	0.00	0.00	0.33	0.00	0.00
	CsODD-C28	180.45	97.47	72.13	180.45	51.62	117.69
	CsODD-C45	1.71	0.00	0.39	1.71	0.00	0.00
	CsODD-C55	1.69	0.43	0.17	1.69	0.88	1.01
	CsODD-C57	269.95	36.77	73.44	269.95	30.66	38.95
	CsODD-C59	14.81	4.14	5.09	14.81	6.71	8.98
	CsODD-C65	0.10	0.00	0.00	0.10	0.00	0.00
	CsODD-C71	290.98	62.98	103.34	290.98	49.35	72.85

CsODD-C73	7.50	1.32	3.37	7.50	3.65	3.93
CsODD-C75	8.84	0.90	0.72	8.84	2.21	3.50
CsODD-C81	41.47	5.45	4.55	41.47	19.52	12.08
CsODD-C84	290.98	62.98	103.34	290.98	49.35	72.85
CsODD-C85	290.98	62.98	103.34	290.98	49.35	72.85
CsODD-C87	2.22	0.26	0.53	2.22	0.31	0.36
CsODD-C88	2.22	0.26	0.53	2.22	0.31	0.36
CsODD-C90	13.72	2.40	5.18	13.72	1.28	1.15
CsODD-C91	1.12	0.70	0.37	1.12	0.35	0.72
CsODD-C94	1.82	0.00	0.00	1.82	0.45	0.32
CsODD-C98	13.05	4.58	7.10	13.05	7.90	7.53
