# Supplementary material for: Genome-Wide Identification of 2-Oxoglutarate and Fe (II)-Dependent Dioxygenase (2ODD-C) Family Genes and Expression Profiles under Different Abiotic Stresses in Camellia sinensis (L.)
Source: Plants (Basel). 2023 Mar 14;12(6):1302. doi: 10.3390/plants12061302 (PMC10051519; doi:10.3390/plants12061302)
Supplement: Supplementary file 1 [file plants-12-01302-s001.zip › TableS8.pdf]

Table S8 The same expression pattern of *CsODD-C* genes under MeJA, PEG and NaCl treatments

Type	Genes	MeJA			PEG			NaCL		
		0 h	24 h	48 h	0 h	24 h	48 h	0 h	24 h	48 h
**Type 3**	CsODD-C36	1.09	4.85	3.08	12.98	116.03	58.71	12.98	93.77	66.43
**Type 4**	CsODD-C21	85.39	56.09	65.27	418.27	163.19	100.93	418.27	110.97	180.80
	CsODD-C109	0.07	0.00	0.00	0.16	0.00	0.00	0.16	0.00	0.00

Note：type3, CsODD-C genes were only upregulated in 48 h after MeJA and PEG treatments；type4, CsODD-C genes were only downregulated in 48 h after MeJA and PEG treatments
