# Supplementary material for: Genome-Wide Identification of 2-Oxoglutarate and Fe (II)-Dependent Dioxygenase (2ODD-C) Family Genes and Expression Profiles under Different Abiotic Stresses in Camellia sinensis (L.)
Source: Plants (Basel). 2023 Mar 14;12(6):1302. doi: 10.3390/plants12061302 (PMC10051519; doi:10.3390/plants12061302)
Supplement: Supplementary file 1 [file plants-12-01302-s001.zip › TableS9.pdf]

Table S9 Protein interaction networks of CsODD-C proteins.

ID_1	Gene_1	ID_2	Gene_2	Combined_score
CsODD-C32	CSS0007745.1	CsODD-C110	CSS0046216.1	0.7
CsODD-C32	CSS0007745.1	CsODD-C151	CSS0018498.1	0.7
CsODD-C46	CSS0045924.1	CsODD-C110	CSS0046216.1	0.7
CsODD-C46	CSS0045924.1	CsODD-C151	CSS0018498.1	0.7
CsODD-C60	CSS0008204.1	CsODD-C146	CSS0013328.1	0.598
CsODD-C145	CSS0001495.1	CsODD-C72	CSS0031656.1	0.423
CsODD-C145	CSS0001495.1	CsODD-C95	CSS0023813.1	0.423
CsODD-C149	CSS0009248.1	CsODD-C26	CSS0011888.1	0.546
CsODD-C149	CSS0009248.1	CsODD-C44	CSS0007481.1	0.546
CsODD-C149	CSS0009248.1	CsODD-C48	CSS0009221.1	0.546
CsODD-C149	CSS0009248.1	CsODD-C49	CSS0031283.1	0.546
CsODD-C149	CSS0009248.1	CsODD-C50	CSS0019497.1	0.546
CsODD-C34	CSS0008358.1	CsODD-C29	CSS0008117.1	0.758
CsODD-C34	CSS0008358.1	CsODD-C30	CSS0019461.1	0.758
CsODD-C34	CSS0008358.1	CsODD-C31	CSS0009584.1	0.758
CsODD-C34	CSS0008358.1	CsODD-C37	CSS0008107.1	0.758
CsODD-C34	CSS0008358.1	CsODD-C38	CSS0009475.1	0.758
CsODD-C34	CSS0008358.1	CsODD-C60	CSS0008204.1	0.758
CsODD-C59	CSS0039460.1	CsODD-C29	CSS0008117.1	0.758
CsODD-C59	CSS0039460.1	CsODD-C30	CSS0019461.1	0.758
CsODD-C59	CSS0039460.1	CsODD-C31	CSS0009584.1	0.758
CsODD-C59	CSS0039460.1	CsODD-C37	CSS0008107.1	0.758
CsODD-C59	CSS0039460.1	CsODD-C38	CSS0009475.1	0.758
CsODD-C59	CSS0039460.1	CsODD-C60	CSS0008204.1	0.758
CsODD-C145	CSS0001495.1	CsODD-C146	CSS0013328.1	0.426
