# Supplementary figures and images for: Genome-Wide Identification of 2-Oxoglutarate and Fe (II)-Dependent Dioxygenase (2ODD-C) Family Genes and Expression Profiles under Different Abiotic Stresses in Camellia sinensis (L.)
Source: Plants (Basel). 2023 Mar 14;12(6):1302. doi: 10.3390/plants12061302 (PMC10051519; doi:10.3390/plants12061302)

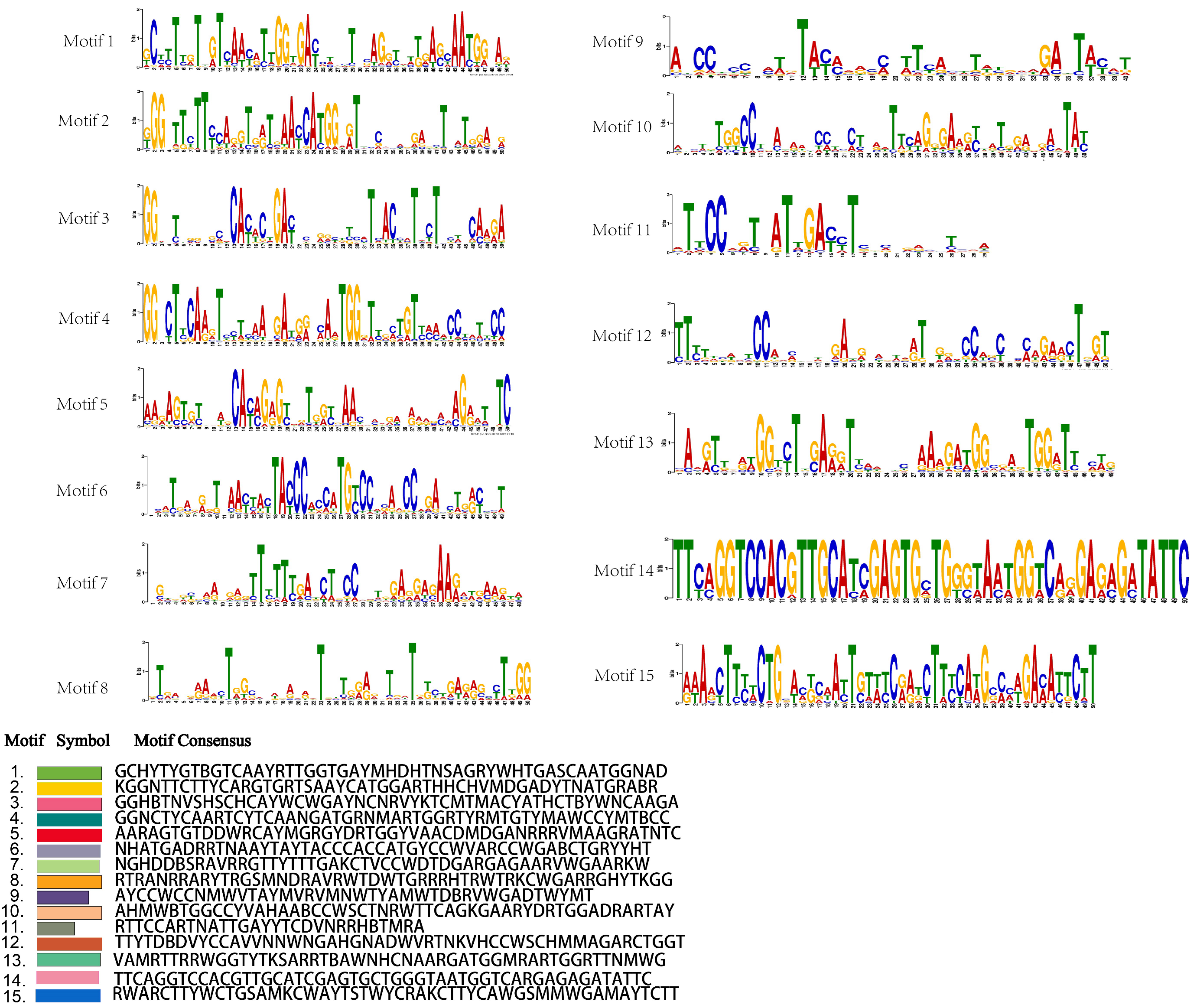

Supplement: Supplementary file 1 [file plants-12-01302-s001.zip › Figure S1.tif]

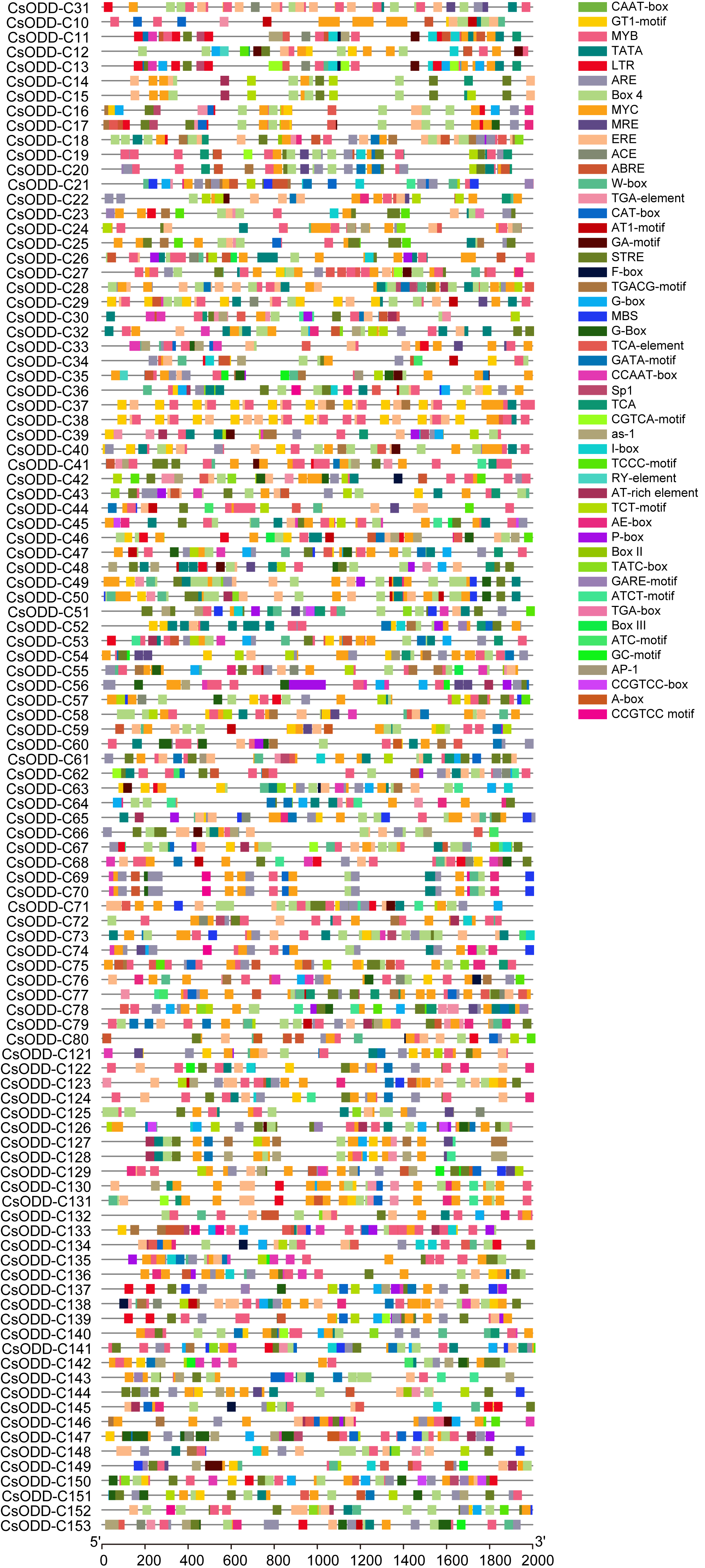

Supplement: Supplementary file 1 [file plants-12-01302-s001.zip › Figure S2.tif]

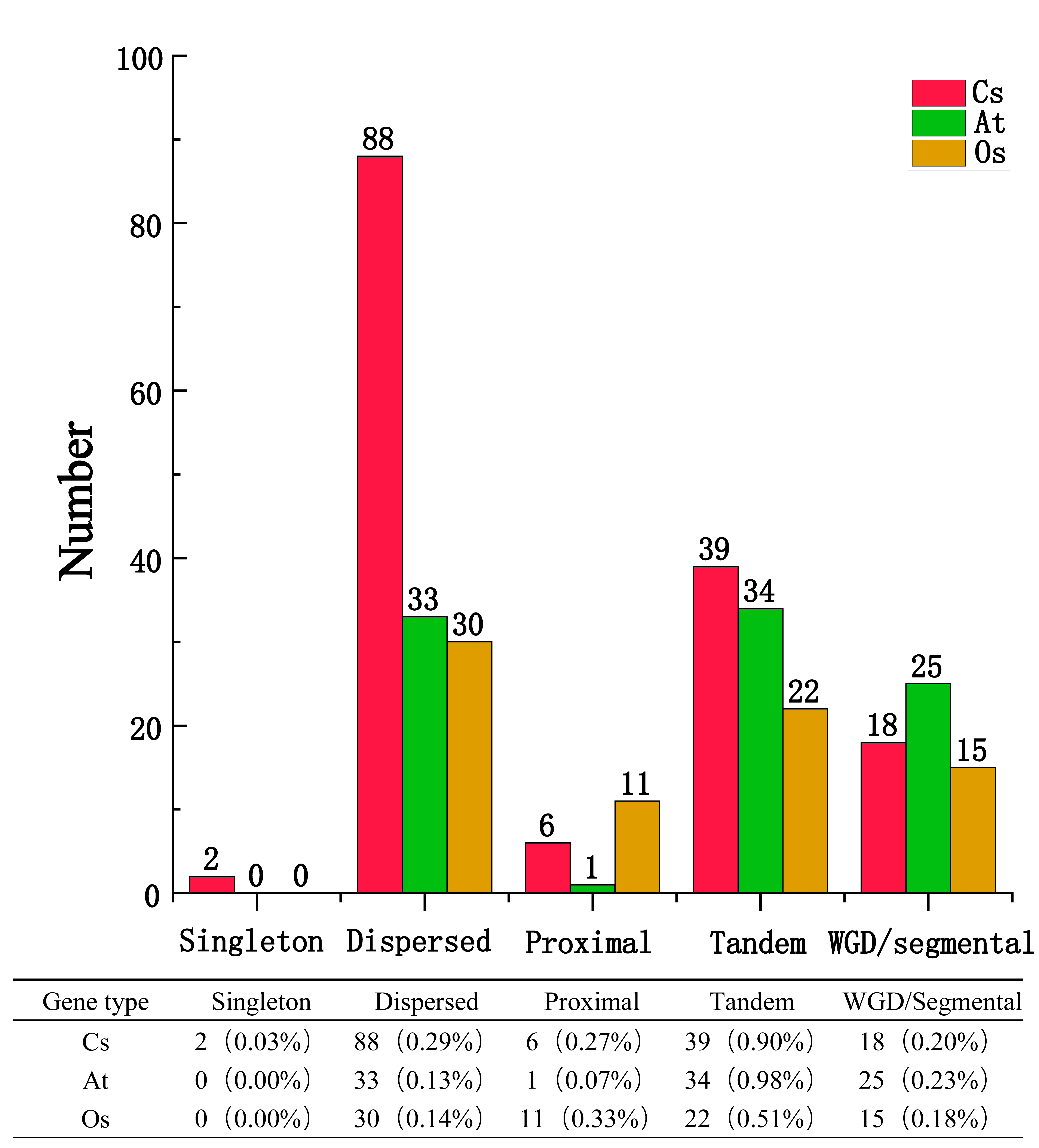

Supplement: Supplementary file 1 [file plants-12-01302-s001.zip › Figure S3.tif]
